# 

**Published:** 1897-12

**Authors:** 


					CONTENTS.
page!
Some Points in Lunacy Practice in
Relation to the General Prac-
titioner.
By J. E. Shaw, M.B. Ed.
301
The Morphology and Pathology of
the Pharyngeal Pouch of Rathke.
By Thomas Carwardine, M.S.
Lond., F.R.C.S. Eng
310
Cases of Hepatic and Intestinal
Surgery.
By Charles A. Morton,
F.R.C.S. Eng.
317
Progress of the Medical Sciences:
Medicine.
R. Shingleton Smith,
M.D.
326
Surgery.
James Swain, M.D.
331
Progress of the Medical sciences
(continued):
Laryngology and Rhinology.
Barclay J. Baron, M.B.
339
Otology.
W. H. Harsant
342
Reviews of Books ..
344
Notes on Preparations for the Sick
361
Meetings of Societies ..
362
The Library of the Bristol Medico-
Chirurgical Society ... ..  367
Local Medical Notes
379
Scraps
383

				

